# Joy at a Vancouver LTSS Facility: Implications for Staff Retention

**DOI:** 10.1093/geroni/igaf122.632

**Published:** 2025-12-31

**Authors:** Lillian Hung, Nathan Boucher, Peter Zhou, Joey Oi Yee Wong

**Affiliations:** University of British Columbia, Vancouver, British Columbia, Canada; Duke University, Durham, North Carolina, United States; The University of British Columbia, Vancouver, British Columbia, Canada; University of British Columbia, Vancouver, British Columbia, Canada

## Abstract

Retaining and empowering caregivers in long-term care (LTC) is critical to sustaining quality care. Despite high demands, long term care staff often receive low wages and limited societal recognition. A positive workplace culture that fosters professional growth, recognition, and well-being is essential for caregiver retention and job satisfaction. This qualitative study explored factors that contribute to staff retention among LTC staff. Four focus groups were conducted with 24 staff, including nurses, care workers, housekeeping, recreation, and rehabilitative staff, in a Canadian LTC home. Data were analyzed thematically to identify key workplace factors influencing staff retention. Three overarching themes emerged: (1) Professional Growth and Development – Staff emphasized the importance of training programs, career advancement pathways, and mentorship in fostering motivation and fulfillment. Continuing education and specialization opportunities contributed to sustained engagement. (2) Recognition and Workplace Culture – Appreciation programs, team cohesion, and a culture of respect and dignity were central to job satisfaction. Feeling valued and included promoted a sense of belonging. (3) Relational Joy in Caregiving – Joy was described as relational and evolving, emerging from supportive environments, positive attitudes, and shared experiences with colleagues and residents. A culture that prioritizes well-being and emotional support reduced burnout and enhanced resilience. In conclusion, retention strategies should be evidence-based, drawing from best practices and ongoing evaluations to measure effectiveness and return on investment. Infusing joy into caregiving strengthens team resilience and fosters compassion, ultimately benefiting both caregivers and residents.

